# 10-Year Trends in Epidemiology, Diagnosis, and Treatment of Cutaneous Leishmaniasis in Hamadan Province, West of Iran (2007–2016)

**DOI:** 10.3389/fpubh.2019.00027

**Published:** 2019-03-05

**Authors:** Azita Akhlagh, Aref Salehzadeh, Amir Hossein Zahirnia, Behrooz Davari

**Affiliations:** Department of Medical Entomology, Hamadan University of Medical Sciences, Hamadan, Iran

**Keywords:** cutaneous leishmaniasis, epidemiology, lorry driver, occupation, West of iran, Hamadan

## Abstract

**Background:** Cutaneous leishmaniasis is one of the most important infectious diseases in eastern Mediterranean countries. The aim of this study was to determine the epidemiological pattern of cutaneous leishmaniasis across a 10-year period in the Hamadan province. This study was considered necessary due to the lack of new information in recent years on the epidemiology of the disease.

**Methods:** This is a descriptive study. The data of patients who were diagnosed with cutaneous leishmaniasis in Hamadan health centers were collected during 10 years (2007–2016) and were analyzed using SPSS software.

**Results:** Of the 908 registered patients, 94% were male and 6% were female. The mean age was 32.7 ± 11.8. About 87.1% of patients had a history of travel to endemic areas. The highest incidence rate was observed in the year 2015 with a rate of 12.6, and the lowest was in 2008 at 1.5 per 100,000 people.

**Conclusion:** Due to an increasing trend in the number of cutaneous leishmaniasis in Hamadan and the effect of occupation, high-risk groups such as lorry drivers should be informed of preventive measures such as using insect repellents. Also, considering the possibility of shaping a new hotspot in the province, thorough reviews and more comprehensive entomological studies are recommended.

## Introduction

Leishmaniasis, a group of parasitic infections, are the third most important arthropod-borne diseases in terms of the global burden of diseases (1). According to the World Health Organization, different forms of leishmaniasis have been reported from 89 countries and over 350 million people are at risk. Based on clinical symptoms, leishmaniasis is divided into cutaneous, mucocutaneous, diffuse cutaneous, and visceral forms. The number of people who suffer from leishmaniasis is estimated to be 12–15 million. Annually, 2 million new cases of leishmaniasis occur, of which approximately 0.5 million are those infected with visceral leishmaniasis and 1.5 million with CL (2). According to reports of WHO, more than two thirds of new cases of cutaneous leishmaniasis (CL) in 2015 occurred in six countries including Brazil, Colombia, Afghanistan, and Iran (3). They are more prevalent in tropical and subtropical regions (4). Cutaneous and visceral forms of the disease are seen mainly in 14 countries of the Eastern Mediterranean Region (5). Phlebotominae sand flies are the main vectors of leishmaniasis and there is a consistent positive correlation between the geographical distribution of sand flies and the disease. The vector breeds in dark places, and as a weak flier feeding usually takes place in close proximity to breeding sites (6). The parasite lives in mononuclear phagocytes of vertebrate host and their uptake by sand flies is following cutting action of vectors (7).

The cutaneous form is the common form of the disease and its history in Iran dates back to about one thousand years ago; the age of Avicenna, an Iranian physician (8). There are two forms of CL in the country: anthroponotic cutaneous leishmaniasis (the urban or dry type) and zoonotic cutaneous leishmaniasis (the rural or wet type) which are caused by *L. tropica* and *L. major*, respectively (9). Despite basic information on the etiology, vectors, and method of transmission of the disease, CL continues to be a serious health problem in Iran, and in some parts of the country, the number of cases is rising (10). They are endemic in 17 out of 31 provinces of Iran (11) and the recorded number of incidences reaches 20,000 new cases every year, although the real number of affected patients is estimated to be 4 or 5 times more than this, as most cases of diseases remain unreported (8).

Previous studies have shown that the distribution of CL in our country varies in different places. Between 2002–2007, Zahirnia et al. carried out an epidemiological survey in Hamadan that indicated the occurrence of about 210 cases of CL in this province; the mean incidence of the disease reported being 2.05/100000 and 85.7% of the patients being between 15 to 44 years old (12). So far 12 species of sand flies have been reported in Hamadan, six of which are known or suspected vectors of leishmaniasis, although none of the dissected phlebotomine has shown leishmanial contamination (9).

All of the above-mentioned factors, along with the lack of precise information on the status of CL in the Hamadan province in recent years, invoke the need for a thorough investigation and precise planning in order to prevent the emergence of new foci of disease in this area. The present study is a descriptive, observational, retrospective study using secondary data obtained from Hamadan province health centers aiming to elucidate the trend of CL in the province.

## Materials and Methods

### The Study Area

**Figure 1:**
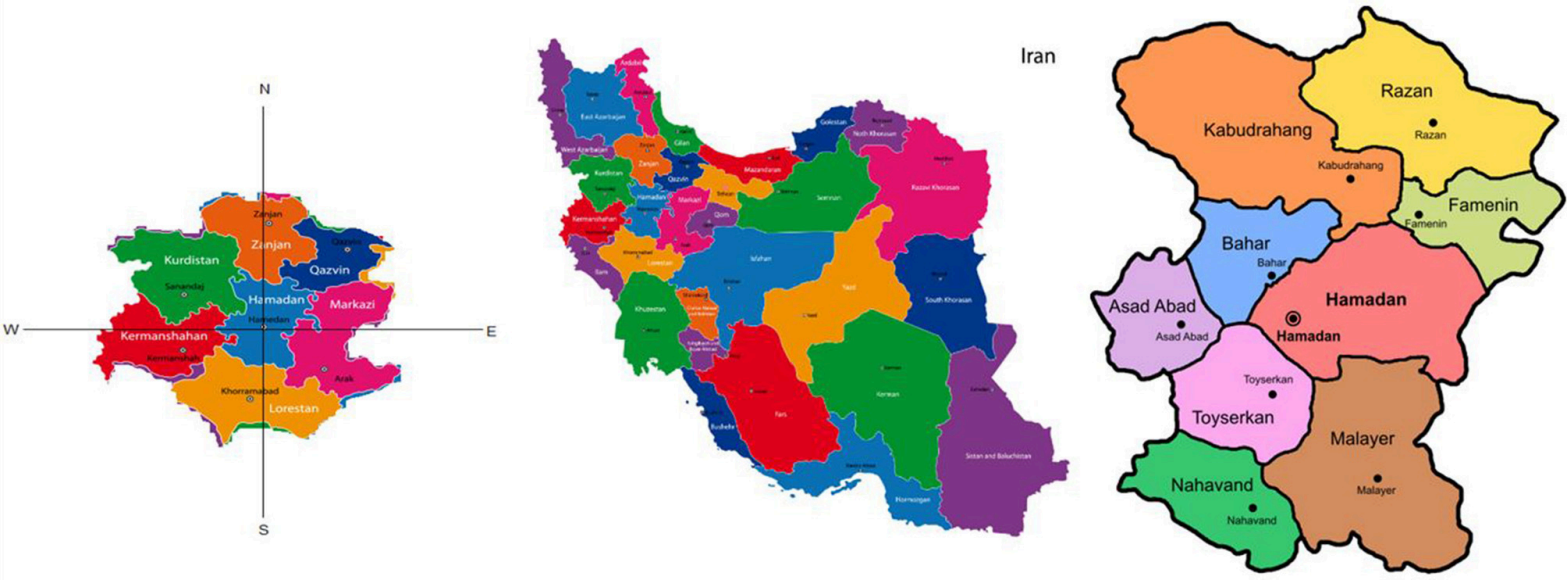
Geographical maps of Hamadan province.

Hamadan is one of the western provinces of Iran. The area of this province is 19,493 square kilometers; it is located among 6 other provinces namely, Zanjan, Qazvin, Lorestan, Markazi, Kurdistan and Kermanshah provinces (Figure 1). The province is between 33°59′-35°48′ N and 47°34′-49°36′ E; it includes 9 counties, 25 districts, 30 towns, and 1,120 villages.

### The Population of the Study Area

According to the census of 2016, the population of this province is 1,738,234 people, of which 1,097,217 are urban and 639,005 are rural populations. The most populated city is the Hamadan in the center and the most sparsely populated city is Famenin in North West of Hamadan.

In general, the province's weather is highly variable as a result of high mountains, rivers and distance from the sea. The winters of this province are cold, snowy and rainy and in the summer temperature is moderate. The trend of weather change in recent years has caused climate change in this province, so that the average minimum and maximum temperature have increased. In addition, reduced rainfall and rising wind speed have also been very effective in making these changes.

### Research Method

The present study is a retrospective study of CL across 10 years in the Hamadan province. All patients diagnosed with cutaneous leishmaniasis in health centers of the Hamadan province from 2007 to 2016 were enrolled. The clinico-epidemiological details of patients were collected using medical records from health centers (including questionnaire and clinical records) of the Hamadan province and data available on the portal of Ministry of Health and Medical Education of Iran (MOHME). Briefly, data encompassed information such as age, gender, wound count, the location of the lesion, the month of affection, location and occupation of the patients at the time of acquiring the infection, travel history during the year before diagnosis, and the treatment method for each individual.

The data also included clinical symptoms, the date of diagnosis (when symptoms and signs were first apparent) and the method of treatment. SPSS software was used for analyzing the collected data and results were drawn up in the form of tables and charts. The incidence rates were calculated using the latest census data. All the procedures were approved by the Research Committee of Hamadan University of Medical Sciences, Iran (Res: IR.UMSHA.REC.1396.493).

## Results

### Number of Cases in Different Cities

**Figure 2:**
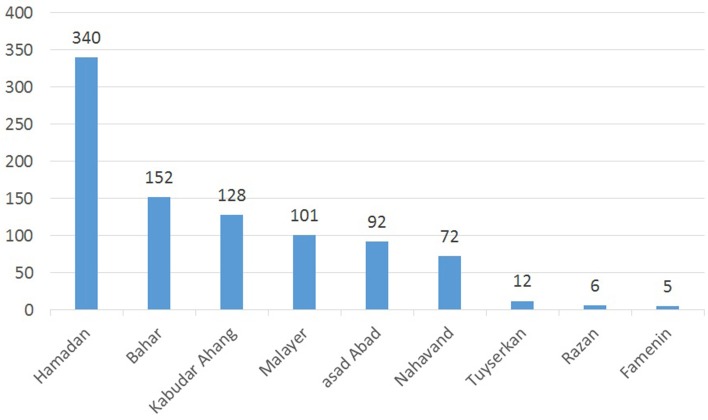
Geographical distribution of cutaneous leishmaniasis in Hamadan province, 2007–2016.

From 2007 to 2016 a total of 908 laboratory-confirmed cases of cutaneous leishmaniasis were reported. A gradual increase in CL incidence has been reported in most counties of the studied areas. Hamadan, with 338 (37.2%) cases of CL, was the most infected city. This figure was followed by Bahar, Kaboudar-Ahang, Malayer, Asad Abad, Nahavand, Razan and Famenin, each with 16.7, 14/7, 11, 10, 7/9, 1/3, and 0.5% cases of CL, respectively (Figure 2).

The annual incidences of CL in the province during the past 10 years are shown in Table 1. The lowest and highest incidences were seen in 2008 and 2015, respectively. As the data show, there is an increasing trend in the incidence of CL from 2007 to 2016.

**Table 1:** The incidence of cutaneous leishmaniasis in the Hamadan province, Iran, 2007–2016.

**Year**	**Incidence rate (per one hundred thousand)**
2007	1.57
2008	1.5
2009	2.08
2010	3.09
2011	3.75
2012	5.09
2013	2.98
2014	9.46
2015	12.6
2016	9.20

**Figure 3:**
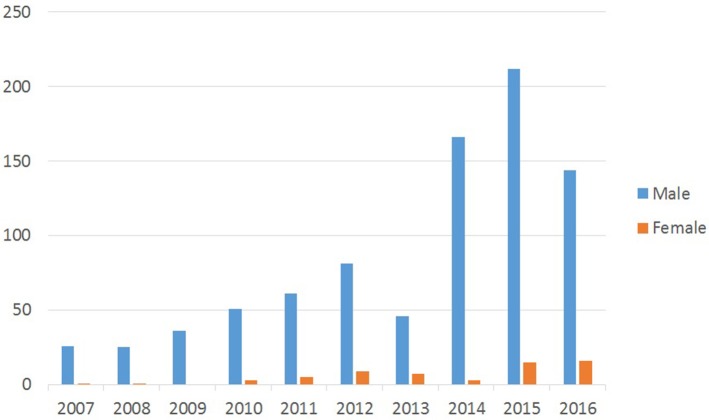
Gender distribution of cutaneous leishmaniasis in Hamadan province during 2007–2016.

Gender distribution of patients has been presented in Figure 3. Out of 908 CL cases, 855 or 94% were male and only 53 cases or 6% were female. The statistical analysis showed a significant difference in the number of CL cases in men in comparison to women (*P* < 0.001).

The cases of disease in the 10-year period sorted by age group are summarized in Table 2. The mean age (± SD) of the patients was 32.7 ± 11.8 years. The highest number of affected people were in the age groups 20–29 years (31.8%), followed by age group 30–39, and the least affected age groups were the 0–9 and 70–79 age groups. There was a significant relationship between the disease and the age group (*P* < 0.001).

**Table 2:** Age distribution of reported cutaneous leishmaniasis cases, Hamadan, province Iran, 2007–2016.

**Age group**	**Year of infection**										
	**2007**	**2008**	**2009**	**2010**	**2011**	**2012**	**2013**	**2014**	**2015**	**2016**	**Percent**
0–9	0	1	0	0	1	2	5	0	4	2	1.65
10–19	1	2	2	2	7	5	5	15	25	10	8.15
20–29	8	10	18	31	37	27	14	51	52	40	31.8
30–39	11	5	5	10	10	32	15	61	70	48	29.4
40–49	5	4	9	4	8	8	7	30	45	26	16.1
50–59	2	3	0	4	2	12	5	10	22	20	8.8
60–69	0	1	2	2	1	2	1	1	4	24	3.1
70–79	0	0	0	1	0	1	1	1	5	0	1
Total	27	26	36	54	66	90	53	169	227	160	908

Regarding living place, before contracting the disease, 486 (53.6%) of the people were living in the urban areas of the province and 422 people (46.4%) in rural areas. The findings indicate that there is no meaningful relationship between the place of residence and the number of patients. In terms of the number of wounds and site of ulcer, the record of patients can be categorized in Table 3.

**Table 3:** Number and percentage of lesions in patients with cutaneous leishmaniasis in Hamadan province during 2007–2016.

**Wound count**	**Count**	**Percent**
1–3 wound(s)	622	68.5%
4–6 wounds	176	19.38%
6 > wounds	110	12.11%

The calculations showed that the average number of lesions was equal to 3.1. The most common site of ulcer was the wrists of patients, making up about 24.3% of all lesions, followed by ankles (20.9%) fore-arm (14.3%) and legs (9.4%). About 7.3% of the lesions were on the trunk while the neck showed the lowest number of lesions (Table 4).

**Table 4:** Body site distribution of lesions in patients with cutaneous leishmaniasis in Hamadan province, 2007–2016.

**Wound location**	**2007**	**2008**	**2009**	**2010**	**2011**	**2012**	**2013**	**2014**	**2015**	**2016**	**Total**
Wrist	7	7	8	21	16	26	16	38	45	37	221
Ankle	4	5	6	13	14	11	12	41	46	38	190
Forearm	4	3	4	2	8	22	9	21	27	30	130
Leg	3	4	5	7	7	8	3	18	22	8	85
Arm	3	3	6	4	6	3	4	17	36	16	98
Face	2	0	1	0	7	11	6	5	20	14	66
Trunk	3	3	6	5	6	3	2	17	14	7	66
Neck	0	1	0	0	2	5	1	9	5	4	27
Scattered	1	0	0	2	0	1	0	3	12	6	25
Total	27	26	36	54	66	90	53	169	227	160	**908**

Seasonal distribution of cutaneous leishmaniasis in Hamadan showed a higher frequency of cutaneous leishmania cases in fall and winter with 342(38%) and 287(32%) cases respectively (the figure is not shown).

Table 5 presents the occupation of patients. As the table shows, more than 53% of patients were drivers while farmers constituted only 3.8% of patients. Statistical analysis showed that there was a correlation between the occupation of people and the occurrence of CL *P* < 0.001.

**Table 5:** Number and percentage of cases of cutaneous leishmaniasis based on occupation Hamadan province during the years 2007–2016.

**Job**	**Count**	**Percent**
Lorry driver	484	53.3
Worker	131	14.4
Soldier	71	7.8
Military	43	4.7
Farmer	35	3.8
Housewife	41	4.5
Student	29	3.2
Employee	12	1.3
University student	11	1.2
Other	51	5.7

### Treatment of Cutaneous Leishmaniasis

According to the guideline, which is issued by the Iranian Ministry of Health, meglumine antimonate is used for treatment of CL. According to documents of Hamadan province health centers, in studied areas during 2007–2016, about 75.8% of patients were treated locally, 23.8% were offered systemic treatment and the rest did not receive any treatment.

### Diagnosis

The diagnosis was based on clinical context and laboratory confirmation. Regarding the diagnostic method, available data showed that 576 (63.4%) of the patients were diagnosed using microscopic methods, finding Leishman body in patient smears; 327 (36%) of them were examined and diagnosed according to clinical signs and epidemiological history and 5 (6.6%) patients were treated with a negative test.

### The Area of Acquisition of Infection

Table 6 presents the most dominant destinations of Hamadanian patients during the year before contracting CL during 2007–2016. Regarding the history of travel to endemic areas, only 26 of the 908 identified cases did not mention the history of travel to endemic areas during the previous year of acquisition of infection, of which 21 related to the Bahar city in the year 2015.

**Table 6:** The most frequent destinations of Hamadanian patients during the year before contracting cutaneous leishmaniasis, 2007–2016.

**Province**	**Percent**	**Number**
**Ilam**	51.2	467
**Khuzestan**	20.4	185
**Esfahan**	6.5	59
**Fars**	3.2	29
**Khorasan Razavi**	1.9	17
**Kermanshah**	1.7	15
**Semnan**	1.7	15
**Hormozgan**	1.7	15
**Sistan Baluchestan**	1.7	15
**Qom**	1.7	15
**Yazd**	1.7	15
**Tehran**	1.5	14
**Bushehr**	0.9	8
**Iraq**	1.4	13

## Discussion and conclusion

The results of the present study indicated that during the period of 2007 to 2016, the number of CL cases in the Hamadan province has raised steadily, with the exception of 2013 and 2016 (Table 4). It also showed that more than 50% of CL occurred in Hamadan City (Figure 2).

As Figure 3 shows, the disease is more prevalent in males, because males are more involved in outdoor activities, which expose them to vectors of leishmaniasis. Other reasons could be the greater exposure of the body surface of men to the vector bites, because they commonly do not cover their skin as women do. This is similar to results of other studies in Iran and other countries (13, 14).

According to the data of this study, the most infected group was in the 20–29 years age group (31.8%), followed by the 30–39 years age group (29.4%), which indicates the higher infection of the active members of the community. This was supported by the works of other researchers such as Iddawela et al. (15). On the other hand, comparing these age groups with the most infected groups of endemic areas such as Isfahan (central Iran) with most infected age group of 10–15 years old (16) or Shadegan (Southwestern Iran) with most infected age group with acute lesion under 20 years old (17) indicates that in case of age distribution of CL, there is a considerable difference between Hamadan and these endemic areas.

Regarding the locations of the lesion or scars and short mouth parts of sand flies as the main vectors, most wounds were seen in uncovered places of the body, such as wrist and ankle; 24.3 and 20.9% respectively. Studies in Lorestan, Damghan, and Yazd also showed that lesions and scars were most prevalent on uncovered areas (18–20). Indeed, more than 79% of lesions were on limbs that in Iranian lorry drivers (all men) are usually more exposed, which suggests that clothing with more coverage can be helpful in reducing the probability of bites from blood-feeding flies such as phlebotominae.

In terms of the number of lesions, most patients had between 1 and 3 lesions on their limbs, which is consistent with the results of studies conducted by Nilforoushzadeh and Norouzinezhad in the Isfahan province and other parts of Iran (21, 22).

**Figure 4:**
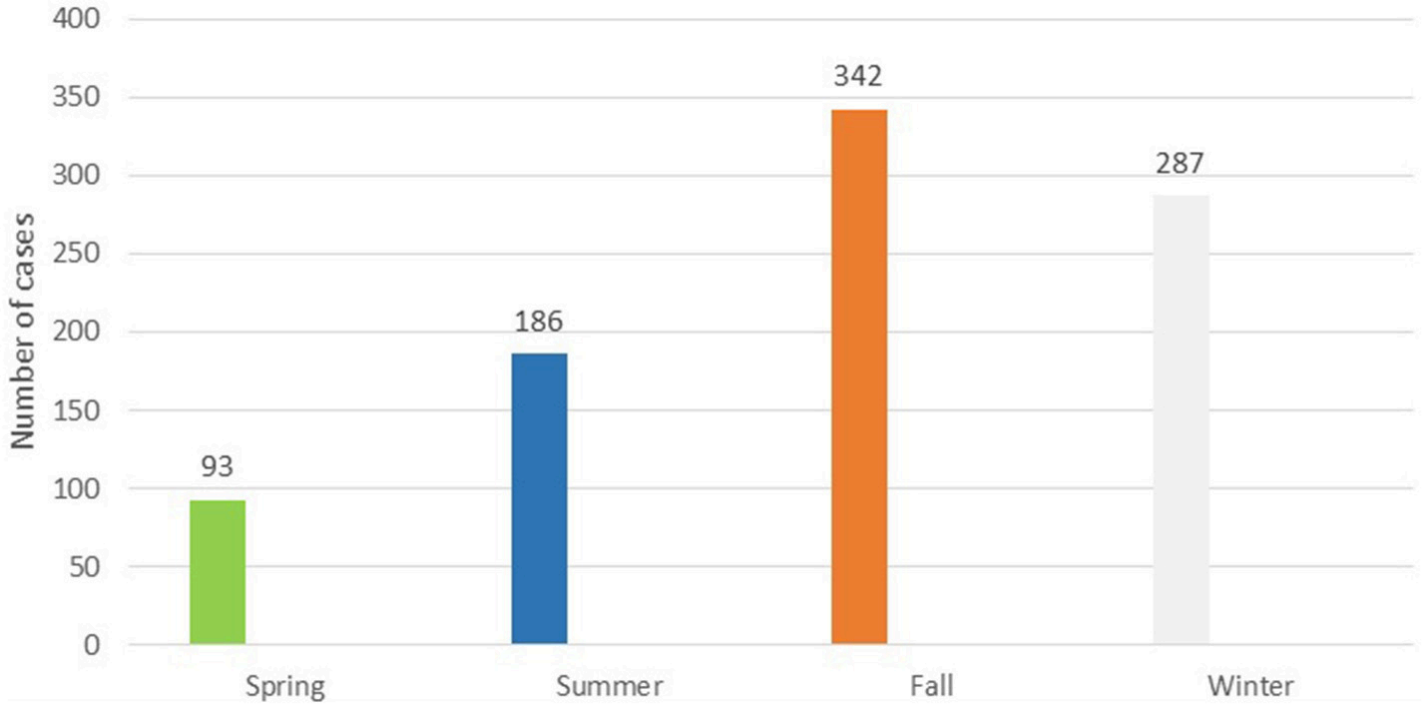
Seasonal distribution of cutaneous leishmaniasis in Hamadan Province during 2007–2016.

Although the outbreak is seasonal (Figure 4), which is consistent with its prevalence in some endemic areas such as Isfahan province (21) with the most frequent abundance of disease in autumn (38.2%) and the least in the spring (10.2%), local transmission in the province has not been confirmed yet and the cases have been assumed as imported cases. Epidemiological data of patients reveal that 87.1% of them had recorded travel to endemic areas in 6 months to 1 year prior to the study. Interestingly, out of 908 patients identified, 26 had no travel history, of which 21 were living in Bahar County in 2015. In this case, further studies are needed to assess vector potentials of sand flies and probable reservoir host of leishmaniasis in this area.

Recent studies have shown that there are outbreaks of disease in areas of the country where the disease was not recorded in the past (23, 24). It should always be remembered that due to the presence of sand flies in most parts of the province (8, 9, 11), the introduction of disease to clean areas is feasible. In addition, a related work has suggested that environmental variation caused by climate change could create new endemic foci in the country (13).

Although comparing the most infected groups of the present study with endemic areas such as Isfahan where the most infected age group is 10–15 years old (16) suggests that at present Hamadan cannot be considered as a focus of CL. Due to the presence of important vectors of leishmaniasis in Hamadan like *Phlebotomus papatasi* and *p. sergenti* (9) and also the presence of infected peoples (12), it is necessary that scientific interventions and precise programs be conducted to prevent the emergence of a new focus in the province. This, in turn, requires important actions such as the identification and thorough treatment of human reservoirs, improving the environmental conditions and the training of risk groups such as drivers, soldiers, and travelers to endemic areas of disease (25).

## Author Contributions

AS and AA conceptualized and designed the study, analyzed the data, and drafted the manuscript. AA, AZ, and BD collected the data. All authors discussed the results and implications and commented on the manuscript at all stages.

### Conflict of Interest Statement

The authors declare that the research was conducted in the absence of any commercial or financial relationships that could be construed as a potential conflict of interest.
